# Incidence and Immunopathology of Myositis in Rectal Cancer Patients Treated With Neoadjuvant Immune Checkpoint Inhibitors and Chemoradiotherapy: Findings From the CHINOREC Trial

**DOI:** 10.1002/mco2.70275

**Published:** 2025-07-07

**Authors:** Rebecca Zirnbauer, Simon Hametner, Jutta Bergler‐Klein, Irene Kuehrer, Askin Kulu, Daphni Ammon, Julijan Kabiljo, Anton Stift, Rainer Schmid, Leonhard Müllauer, Clemens Bittermann, Friedrich Laengle, Klaus Machold, Stephan Blüml, Michael Bergmann, Johannes Laengle

**Affiliations:** ^1^ Division of Visceral Surgery Department of General Surgery Comprehensive Cancer Center Vienna Medical University of Vienna Vienna Austria; ^2^ Division of Neuropathology and Neurochemistry Department of Neurology Medical University of Vienna Vienna Austria; ^3^ Division of Cardiology Department of Medicine II Medical University of Vienna Vienna Austria; ^4^ Department of Radiation Oncology Comprehensive Cancer Center Vienna Medical University of Vienna Vienna Austria; ^5^ Department of Pathology Comprehensive Cancer Center Vienna Medical University of Vienna Vienna Austria; ^6^ Clinical Department of General Visceral and Vascular Surgery University Hospital Wiener Neustadt Wiener Neustadt Austria; ^7^ Division of Rheumatology Department of Medicine III Medical University of Vienna Vienna Austria

**Keywords:** cardiac troponin I, cardiac troponin T, chemoradiotherapy, immune checkpoint inhibitors, myositis, rectal cancer

## Abstract

Myositis is a rare (<1%) but potentially severe immune‐related adverse event (irAE) of immune checkpoint inhibitors (ICIs), with a 40%–50% fatality rate. Its incidence and pathology in curative, neoadjuvant settings, particularly with chemoradiotherapy (CRT), remain poorly defined. Given the severity, stringent diagnostic and therapeutic approaches may be warranted in curative patients. In the CHINOREC trial, 50 rectal cancer (RC) patients receiving neoadjuvant CRT with ipilimumab (IPI) and nivolumab (NIVO) were prospectively monitored for myotoxicity biomarkers, including creatine kinase (CK) and cardiac troponins (cTnT, cTnI). Patients with CK and cTnT levels above the upper limit normal with or without overt clinical symptoms underwent muscle biopsy and guideline‐adapted treatment (glucocorticoids, immunoglobulin, infliximab, plasma exchange). Six patients (12%) developed biopsy‐confirmed myositis. Elevated cTnT, but not cTnI, distinguished skeletal from cardiac involvement, aligning with normal cardiac magnetic resonance imaging (CMR) findings. Immunohistochemistry showed a predominant CD8+ T cell infiltrate and patchy human leukocyte antigen (HLA) Class I upregulation. Despite myositis, all patients underwent successful tumor resection with normalized CK levels and no residual cardiac dysfunction. ICI‐induced myositis may be more frequent in neoadjuvant‐treated RC patients receiving CRT+ICI than in palliative settings. Comprehensive biomarker monitoring and early T cell‐directed intervention are essential for mitigating life‐threatening irAEs while preserving oncologic outcomes.

## Graphical abstract

ICI‐induced myositis occurred at a higher‐than‐expected rate (12%) in neoadjuvant‐treated rectal cancer patients receiving CRT+ICI. Elevated cTnT, but not cTnI, differentiated skeletal from cardiac involvement. Immunopathology showed a predominant CD8+ T cell infiltrate, highlighting a T cell‐mediated mechanism that necessitates early detection and targeted immunomodulation.

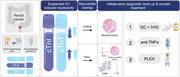

## 1 Introduction

Immune checkpoint inhibitors (ICIs), such as ipilimumab (IPI) and nivolumab (NIVO), have transformed the management of metastatic solid cancers, showing significant therapeutic success [1]. Efforts are now being made to integrate ICIs into the curative neoadjuvant setting [2]. However, caution is warranted as these patients are at risk for a range of immune‐related adverse events (irAEs) that may require aggressive immunosuppression, potentially compromising treatment outcomes [3, 4, 5]. While irAEs affecting the skin or endocrine systems are relatively common and typically have low case fatality rates (CFR) [3], ICI‐induced myositis, though rare with a cumulative incidence of <1%, is potentially life‐threatening if not identified and treated early [6]. Retrospective data from the World Health Organization (WHO) pharmacovigilance database highlight that ICI‐induced myocarditis has the highest CFR among irAEs, estimated at 40%–50% [6, 7].

The true incidence of ICI‐induced myotoxicities is likely underestimated, particularly as ICIs are currently the standard of care (SOC) in palliative settings. Symptoms such as fatigue, weakness, and pain, potential indicators of myotoxicity, are often nonspecific and can mimic the natural course of metastatic disease. Recently, ICI therapy has been extended to adjuvant and neoadjuvant settings, where the aim is curative. While ICI‐induced myositis appears manageable in adjuvant settings with current guidelines (SITC, ESMO, ASCO), its use in neoadjuvant settings poses unique challenges. First, ICIs are often combined with radiotherapy (RT) or chemoradiotherapy (CRT), yet little is known about the incidence of ICI‐induced myositis in this combination. Early‐phase studies in rectal cancer (RC) using CRT and ICIs are showing encouraging results regarding complete remission rates [8, 9, 10]. Second, irAEs must not preclude surgical resection, which is often essential for curative intent. Third, there is little data available about the type of myositis, occurring after ICI inhibition. Consequently, even rare but potentially lethal irAEs, such as myositis, must be carefully managed to prevent progression to severe forms, particularly in settings with high curative potential. Given the limited data, current recommendations for managing ICI‐induced myositis in neoadjuvant settings are sparse.

Due to a lack of robust data, current guidelines for ICI‐induced myositis rely primarily on case reports and series. Myositis grading (G1–G4) and management are based on unspecific symptoms such as “weakness with or without pain” and “creatine kinase (CK) elevation.” Treatment varies from glucocorticoids (GC) to more potent immunosuppressive therapies, such as rituximab (anti‐CD20), often with unsatisfactory outcomes [11, 12, 13, 14]. Delayed diagnosis and nonspecific treatment may contribute to poor prognoses. Distinguishing myocarditis from myositis is also challenging, as both irAEs share similar myotoxicity biomarkers, and while the European Society of Cardiology (ESC) cardio‐oncology guidelines recommend cardiac surveillance during ICI treatment [15, 16, 17], many questions remain unresolved.

In the recent phase II CHINOREC trial, we evaluated the impact of IPI and NIVO in RC patients receiving neoadjuvant CRT, with prospective monitoring of myotoxicity biomarkers. Here, we report the incidence, immunopathology, and treatment outcomes of biopsy‐verified ICI‐induced myositis in this cohort. Our findings underscore an underestimated threat and provide insights that may improve clinical outcomes through early awareness and management.

## 2 Results

### 2.1 Incidence of Myositis in RC Patients Receiving Neoadjuvant ICI With CRT

Between June 2020 and November 2023, 50 patients were assigned to the CRT+IPI/NIVO arm. Six patients (12%) developed biopsy verified myositis. Patient characteristics are shown in Table 1. The median time to myositis onset, calculated from the first ICI dose, was 32 days (95% CI 21–56). Patient 1 had an overlap with myasthenia gravis (MG). Of note, no patient in the control arm (CRT without IPI/NIVO) developed any myotoxicity (*n* = 30). These findings suggest a significantly higher incidence of myositis in the ICI‐treated group compared to controls, warranting further investigation into risk factors and pathophysiology

**TABLE 1:** Patient characteristics.

	ICI‐Myositis	No myositis
**Total No. of patients** ^a^	6	44
**Age, years, median (range)**	65 (49–81)	60 (44–83)
**Sex, no. (%)**		
Male	4 (67)	27 (61)
Female	2 (33)	17 (39)
**Ethnicity**		
European	6 (100)	44 (100)
**BMI, median (range)**	26 (20–41)	26 (18–43)
**Concomitant statins, No. (%)**		
Yes	1 (17)	9 (20)
No	5 (83)	35 (80)
**Time to myositis from first ICI dose, days, median (95% CI)**	32 (21–56)	NA

Abbreviation: NA, not applicable.

^a^ Patients in the IPI/NIVO+CRT arm.

### 2.2 Clinical Course and Treatment of ICI‐Induced Myositis

**FIGURE 1:**
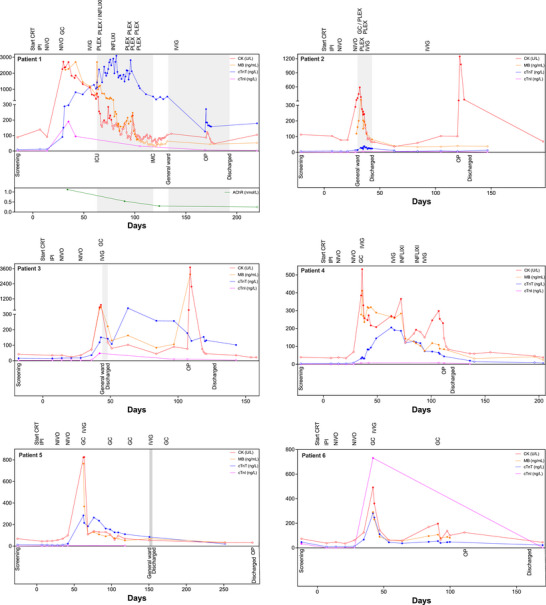
cTnI distinguishes a cardiac overlap in ICI‐induced myositis. Levels of CK, MB, cTnT and cTnI (ordinate) are plotted over time (abscissa). Each data point represents a specific lab value at a specific timepoint, with filled circles indicating values above the respective upper limit of normal (ULN) and empty circles indicating values within the normal range. Treatment sequences and hospitalization events are noted. CK, creatine kinase; MB, myoglobin; cTnT, cardiac troponin T; cTnI, cardiac troponin I; IPI, ipilimumab; NIVO, nivolumab; PLEX, plasma exchange; GC, glucocorticoids; IVIG, intravenous immunoglobulin; CRT, chemoradiotherapy; ICU, intensive care unit; IMC, intermediate care unit; AChR, anti‐acetylcholine receptor.

A detailed clinical case description of all individual ICI‐induced myositis patients is described in the (additional details are available in the Supporting Information). Briefly, among the six patients with biopsy‐confirmed ICI‐induced myositis, myotoxicity biomarkers (CK, cTnT) increased after the second dose of NIVO, prompting clinical evaluation and a stepwise guideline‐adapted (SITC, ESMO, ASCO) myositis treatment, including GC, intravenous immunoglobulin (IVIG), infliximab (INFLXI), and/or plasma exchange (PLEX). While symptoms varied in severity, all patients received GC (1–2 mg/kg) and IVIG (2 g/kg). In two cases, PLEX was necessary due to persistent or worsening symptoms and two patients received INFLIXI (5 mg/kg) to control refractory disease. One patient experienced severe disease progression, requiring intensive care unit (ICU) and prolonged hospitalization, while the remaining cases were managed without ICU admission. Despite myositis, all patients underwent successful oncologic tumor resection. Myotoxicity biomarkers normalized post‐treatment and functional recovery was achieved in most cases. Table 2 provides a summary of the clinical features of ICI‐induced myositis cases, while Figure 1 presents a graphical overview of the treatment strategies alongside the myotoxicity biomarker courses. Overall, stepwise guideline‐adapted therapy was effective in most cases, allowing all patients to proceed with curative‐intent surgery. In addition, a proposed screening and treatment algorithm for ICI‐induced myotoxicities is available in the Supporting Information.

**TABLE 2:** Clinical features of ICI‐induced myositis cases.

Patient no.	1	2	3	4	5	6
**Time to myositis from first ICI dose, days**	21	23	36	28	56	35
**Distribution of symptoms**						
Proximal UE	Yes	No	No	Yes	Yes	No
Proximal LE	Yes	Yes	Yes	Yes	Yes	Yes
Neck extensors	Yes	No	No	Yes	Yes	No
Extraocular	Yes	No	No	No	Yes	No
Bulbar (dysphagia, hoarseness)	No	No	No	No	No	No
Respiratory	Yes	No	No	No	No	No
**Laboratory findings**						
CK >ULN	Yes	Yes	Yes	Yes	Yes	Yes
MB >ULN	Yes	Yes	Yes	Yes	Yes	Yes
cTnT >ULN	Yes	Yes	Yes	Yes	Yes	Yes
cTnI >ULN	Yes	No	Yes	No	No	Yes
Myositis antibodies^e^	No	No	No	No	No	No
**Examinations**						
Electrocardiogram (ECG) normal	Yes	Yes	Yes	Yes	Yes	Yes
Transthoracic echocardiogram (TTE) normal	Yes	Yes	NA	Yes	Yes	Yes
Cardiac magnetic resonance imaging (CMR) normal	Yes	NA	Yes	Yes	Yes	Yes
Coronary computed tomography angiography normal	Yes	NA	NA	NA	Yes	Yes
Electromyography (EMG) normal	Yes	NA	NA	NA	Yes	NA
**Overlap syndromes**	Yes	No	No	No	No	No
Myasthenia (anti‐AChR positive)	Yes	No	No	No	No	No
Peripheral sensory neuropathy	No	No	No	No	Yes	No
Bell's palsy	No	No	No	No	Yes	No
Hepatitis	No	No	No	No	Yes	No
Myocarditis	No	No	No	No	No	No
**Myositis treatment**						
Glucocorticoids (GC)	Yes	Yes	Yes	Yes	Yes	Yes
Intravenous immunoglobulin (IVIG)	Yes	Yes	Yes	Yes	Yes	Yes
Infliximab (INFLIXI)	Yes	No	No	Yes	No	No
Plasma exchange (PLEX)	Yes	Yes	No	No	No	No
**Myositis outcome**						
Resolved^a^	Yes	Yes	Yes	Yes	Yes	Yes
**Muscle histopathology**						
Necrotic fibers / myophagia^d^	+++	++	+	+	+	+
Perifascicular atrophy	0	0	0	0	+	0
Regenerative fibers	++	+	0	0	++	+
Endomysial inflammatory infiltrates	+++	++	+	+	+	+
Perimysial inflammatory infiltrates	0	0	0	+	0	0
CD4 presence	+	+	+	+	+	0
CD8 presence	++	++	++	++	+	+
CD8 intact fiber invasion	++	+	0	0	0	0
B cell presence (CD20/CD79a)	0	0	0	0	0	0
HLA class I sarcolemmal overall	+++	++	+	++	++	++
HLA class I perifascicular	0	0	0	0	+	0
c5b9 capillaries	0	0	0	0	0	0
c5b9 sarcolemmal	0	+	0	+	0	+
Additional pathological features	NA	NA	^b^	^c^	NA	NA

Abbreviations: AChR, acetylcholine receptor; CK, creatine kinase; cTnI, cardiac troponin I; cTnT, cardiac troponin T; HLA, human leukocyte antigen; LE, lower extremities; MB, myoglobin; NA, not applicable; UE, upper extremities; ULN, upper limit normal.

^a^ age‐appropriate instrumental and self‐care activities of daily living (ADL).

^b^ mild neurogenic atrophy.

^c^ mild fiber type 2 atrophy.

^d^ Myophagia is defined as phagocytosis of necrotic muscle fibers by immune cells.

^e^ Myositis antibodies tested include Mi‐2α, Mi‐2β, TIF1γ, MDA5, NXP2, SAE1, Ku, PM‐Scl100, PM‐Scl75, Jo1, SRP, PL‐7, PL‐12, EJ, OJ and Ro‐52.

### 2.3 cTnI Distinguishes a Cardiac Overlap in ICI‐Induced Myositis Compared to cTnT

Individual cardiac (cTnT, cTnI) and skeletal muscle biomarkers (CK, MB) for each patient are shown in Figure 1. Elevated cTnT and CK values above their respective upper limits of normal (ULN) signaled the onset of initially mild or minimal symptomatic ICI‐induced myositis. Over time, MB levels demonstrated a strong correlation with CK levels. Following the initiation of myositis treatment, CK and MB levels declined, indicating a positive treatment response, whereas cTnT levels exhibited a delayed peak and slower decline. Interestingly, cTnT levels in some cases remained above ULN even after CK and MB levels had normalized. Notably, cTnI levels were almost universally normal across patients, effectively ruling out cardiac involvement based on indirect assessments, including cardiac magnetic resonance imaging (CMR), transthoracic echocardiogram (TTE), or electrocardiogram (ECG).

NT‐proBNP did not correlate with cTnT, CK, or MB levels (data not shown). This lack of correlation may partly be due to iatrogenic changes induced by myositis treatments (i.e. IVIG), which can lead to intravascular overload and subsequently, physiological but clinically irrelevant NT‐proBNP elevations. These findings highlight cTnI as a reliable marker for distinguishing ICI‐induced skeletal myositis from cardiac involvement, reducing the need for unnecessary cardiac evaluations.

### 2.4 ICI‐Induced Myositis Is Primarily Mediated by Cytotoxic T Cells

**FIGURE 2:**
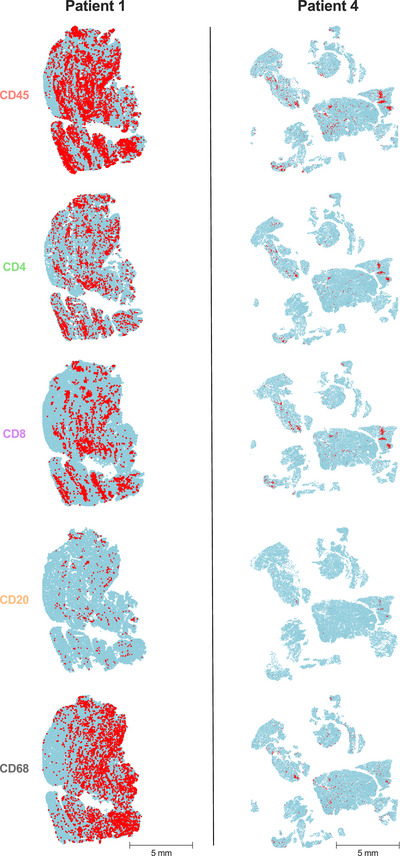
Whole‐slide tissue example of muscle immune cell infiltration. Whole‐slide muscle sections of ICI‐induced myositis patients were stained by IHC for pan leucocytes (CD45), cytotoxic T cells (CD8), T helper cells (CD4), B cells (CD20) and macrophages (CD68). Representative example of patient 1 and 4 is shown. Filled blue circles represent all cell nuclei and filled red circles characterize positive stainings for each specific antibody. Scale bar 5 mm.

**FIGURE 3:**
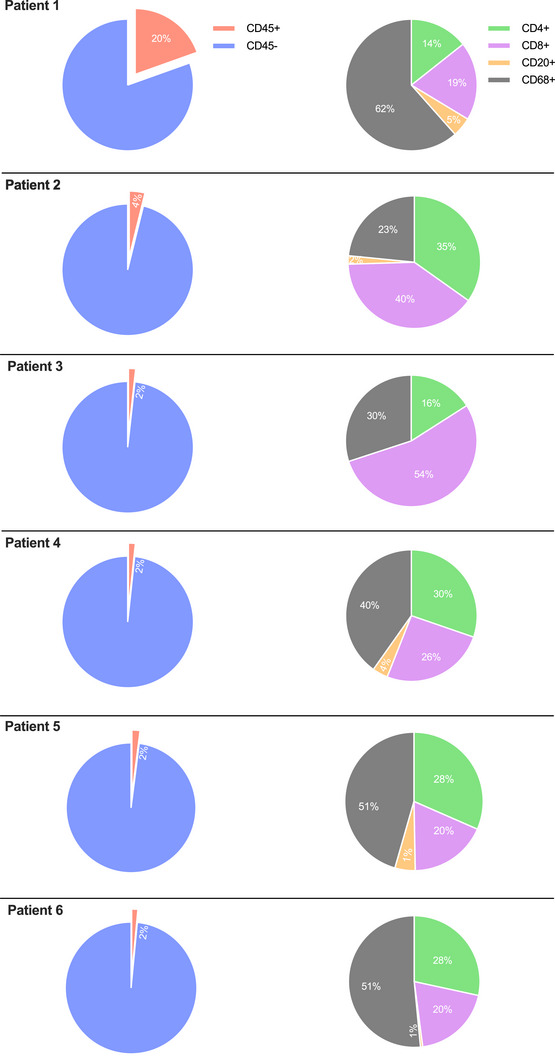
Immune cell composition in ICI‐induced myositis muscle biopsies. Absolute immune cell counts of whole‐slide muscle tissues are portrayed as fractions using pie charts. Percentages of pan‐leukocytes (CD45) of all cell nuclei is depicted in the left pie charts. Further percentage distribution of cytotoxic T cells (CD8), T helper cells (CD4), B cells (CD20) and macrophages (CD68) out of all CD45 positive cells is illustrated in the right pie charts.

The immune cell infiltrate of muscle biopsies was phenotypically characterized by immunohistochemistry (IHC). Examples of whole‐slide images for pan‐leucocytes (CD45), cytotoxic T cells (CD8), T helper cells (CD4), B cells (CD20), and macrophages (CD68) are presented in Figure 2. Immune cells were further absolutely quantified using a computational approach (Figure 3). Patient 1, who experienced the most severe clinical course (including ICU admission and intubation), demonstrated a dense leukocyte infiltrate, with macrophages representing the most abundant population (62%). In contrast, the remaining patients showed lower macrophage proportions (range 23%–51%) and less pronounced muscle fiber necrosis or myophagia. While histiocytes were numerically dominant in most samples, the majority of patients exhibited a T cell‐enriched infiltrate (range 48%–75%), with cytotoxic T cells (CTLs) forming the largest lymphocyte subset (range 18%–54%). B cells were nearly absent across all samples (range 0%–5%). These findings suggest a prominent involvement of CD8+ T cells in the inflammatory response, although the contribution of macrophages and other cell types should not be underestimated. The observed profile contrasts with B cell‐mediated autoimmune myopathies and may reflect a distinct immunopathogenic mechanism in ICI‐associated myositis.

### 2.5 Myofiber Necrosis Correlates With CTL Infiltration

Histopathologic findings of muscle biopsies are shown in Table 2. While all six patients displayed a similar inflammatory pattern, the overall degree of muscle involvement varied substantially. Patient 1, with the most severe muscle involvement, exhibited endomysial infiltrates dominated by CD8+ T cells, which invaded intact myofibers and were associated with patchy upregulation of HLA class I around inflammatory foci. Notably, no terminal complement complex (C5b9) deposits were observed. Patient 2 displayed similar, albeit less intense, involvement, with minor granular complement deposits along the sarcolemma. Patient 3 showed only weak HLA class I upregulation, a few infiltrating T cells, and mild neurogenic atrophy, possibly unrelated to inflammation. Patient 4 exhibited moderate CD8+ T cell infiltration and HLA class I upregulation within the endomysium. Patient 5 demonstrated focal perifascicular atrophy and mild HLA class I upregulation in atrophic fibers. Patient 6 showed minimal inflammation but still presented with patchy HLA Class I upregulation and CD8+ T cell infiltration.

Mitochondrial pathology, such as COX‐negative or ragged‐red fibers, was virtually absent across all samples. Similarly, rimmed vacuoles and coarse capillary complement deposits were absent. Overall, the extent of myofiber necrosis and myophagia, defined as the phagocytosis of necrotic muscle fibers by immune cells, appeared to correlate primarily with the degree of endomysial CD8+ T cell infiltration, direct invasion of intact myofibers, and patchy sarcolemmal HLA class I upregulation. Perifascicular atrophy and HLA class I upregulation were observed in only 1 of the 6 cases. The degree of myofiber necrosis appears to be directly associated with CD8+ T cell infiltration and sarcolemmal HLA class I upregulation, suggesting a CTL‐mediated mechanism of muscle injury.

## 3 Discussion

ICI‐induced myotoxicities, including myositis, have traditionally been considered rare irAEs, even across various ICI combinations [6, 7]. In this study, we observed a 12% incidence of ICI‐induced myositis in RC patients treated with neoadjuvant CRT and ICIs. This incidence exceeds previously reported rates in RC, where two studies of neoadjuvant RT and ICI in RC reported myositis and myocarditis rates of 4.4% and 4.5%, respectively [8, 9]. Importantly, these myotoxicities were not present in control arms lacking ICIs, indicating a potential association between ICI use and increased myotoxic risk. A hypothesis‐generating explanation for the elevated incidence observed here may involve immunogenic cell death (ICD) induced by radiation in the muscles surrounding the rectal tumor, such as the pelvic and gluteal regions. This local immunogenic environment, when combined with ICIs, might stimulate a muscle‐specific, T cell‐mediated immune response. While radiation‐induced myositis is rarely reported with RT alone, similar mechanisms have been described in cases of radiation recall myositis [18].

Currently, there is no consensus on preemptive screening for ICI‐induced myotoxicities, particularly in neoadjuvant CRT settings. In our study, we screened patients using CK and cTnT, following muscle biopsy upon biomarker elevation. Elevated biomarkers consistently correlated with biopsy‐confirmed myositis, even in mild or minimal symptomatic cases, underscoring the value of early screening. Given the high curative potential of CRT/ICI combinations for RC [8, 9, 10], it is likely that these regimens will see broader application in the future. This necessitates vigilance for irAEs with potential lethality, such as myositis, as illustrated by one of our patients (Patient 1) who required mechanical ventilation. Thus, we recommend reconsidering the approach to monitoring for this type of irAE in the neoadjuvant setting. A retrospective cohort study identified concurrent statin therapy and ICI treatment as a potential risk factor for developing myotoxicities [19]. However, in our study, only one patient was receiving statins during ICI treatment. Notably, we did not assess 3‐hydroxy 3‐methylglutaryl coenzyme‐A reductase (HMGCR) antibodies in this patient (Patient 1), leaving it unclear whether statin‐associated IMNM contributed to the disease course.

The timing of myositis treatment initiation remains an important consideration. In routine settings, mild, nonspecific symptoms of ICI‐induced myositis are often overlooked, especially in palliative or adjuvant contexts. However, our observations suggest that early intervention in neoadjuvant settings may prevent severe outcomes, following the principle that “time is muscle”. This approach applied to five of our six patients, allowing for timely myositis management without delaying surgery. The challenge of balancing the risks of over‐diagnosis and over‐treatment against the need for timely intervention in potentially life‐threatening irAEs, as illustrated by Patient 1, remains a significant issue in clinical practice.

Regarding the type of myositis treatment, we followed guidelines from SITC, ESMO, and ASCO, with adjustments based on our findings. We prioritized GC and T cell‐focused immunosuppression based on biopsy discoveries that suggested a CD8+ T cell‐mediated pathology, distinct from conventional B cell‐mediated myositis (PM or DM). Our biopsy findings align with recent work by Pinal‐Fernandez *et al.*, which identified three histopathological subtypes of ICI‐induced myositis, namely, inflammatory myositis, necrotizing myositis, and a DM‐like subtype, based on transcriptomic and pathological features [20, 21]. While our cases did not display DM‐like characteristics, they shared overlapping features of both inflammatory and necrotizing myositis, supporting the heterogeneity of ICI‐induced muscle injury. The absence of autoantibodies and features, such as perifascicular atrophy, guided us away from B cell‐targeted therapies. Early, high‐dose GC administration is supported by studies on ICI‐induced myocarditis [22, 23], and selective immunosuppressive agents have shown efficacy in ICI‐induced colitis [24]. In our cohort, moderate‐dose GC (1 mg/kg) was administered with IVIG to reduce reliance on higher GC doses. Tumor necrosis factor alpha (TNF‐α) inhibitor was introduced in cases where initial treatments proved insufficient, given its minimal side effects, multiple immunomodulatory effects, and potential to support T cell‐mediated antitumor immunity without significant impact on oncologic outcomes [25, 26, 27, 28, 29]. In refractory cases, PLEX was applied for additional immunomodulation without broad immunosuppression, although it required central venous access.

Distinguishing myositis with cardiac involvement remains a diagnostic challenge. Our data suggest that elevated cTnT with normal cTnI strongly indicates skeletal muscle involvement without cardiac overlap. Although we did not perform endomyocardial biopsies, non‐invasive assessments, including CMR, TTE, and ECG, supported this finding, consistent with prior studies in which elevated cTnT levels were found in skeletal myopathies without corresponding cTnI elevation [30, 31, 32]. This biomarker pattern helps avoid unnecessary cardiac assessments and aligns with studies suggesting that *TNNT2* (mRNA encoding for cTnT) re‐expression in skeletal muscle can lead to elevated cTnT without myocardial involvement [33]. It has been noted that CMR can be falsely negative if performed too soon (<4 days) after patient admission and cTnT might be persistently elevated despite clinical remission of ICI‐induced myositis [34, 35].

Study limitations include the small sample size and the absence of a control group without myositis treatment. Nonetheless, our study offers a unique contribution, providing a homogeneous patient cohort treated with a standardized neoadjuvant ICI+CRT, prospective biomarker assessment, a comprehensive diagnostic work‐up focusing on myotoxicity (i.e. muscle biopsy, CMR, autoantibodies, etc.). This allows for valuable insight into the course of ICI‐induced myositis in a curative treatment setting. Notably, electromyography (EMG) data was lacking for most patients. While EMG could have provided additional electrophysiological characterization, all cases were biopsy‐confirmed, with clear clinical and biomarker evidence of myositis. Nevertheless, future studies should incorporate EMG as part of a standardized diagnostic work‐up to better delineate neuromuscular involvement.

In conclusion, our findings suggest that the relatively high incidence of myositis in patients receiving CRT with ICI warrants comprehensive diagnostics for early detection and intervention. The necessity of early treatment initiation requires validation in larger trials, which will be challenging due to the rarity of ICI‐induced myositis and the diversity of ICI regimens and tumor types. In addition, if a control arm for ICI‐induced myositis receiving no treatment is ethically feasible and needs to be discussed by an IRB. For now, treatment for ICI‐induced myositis should be guided by clinical judgment, with biopsy‐confirmed diagnosis offering a more targeted approach to care.

## 4 Materials and Methods

### 4.1 Study Design

The study design of the CHINOREC trial (ClinicalTrials.gov identifier NCT04124601) was reported previously [36, 37, 38]. It was a prospective, randomized, open‐label, multicenter, phase II investigator‐initiated trial (IIT). In total, 80 patients with RC are randomly assigned (randomization ratio 30:50) to receive either neoadjuvant CRT alone or concomitantly with a single dose of IPI, following three cycles of NIVO in a sequential approach. Randomization was done in a permuted block design with a block size of 8. Patients underwent surgical resection in a curative intent within 10–12 weeks post‐CRT (Figure S1). The primary endpoint was the safety of neoadjuvant IPI and NIVO concomitant to CRT, following surgical resection. The consort flow diagram is shown in Figure S2. All planned 80 patients have been enrolled (cut‐off date 2023‐11‐15).

### 4.2 Patients

Patients (>18 years of age) with a histologically confirmed carcinoma of the rectum are eligible if there is a medical need for a neoadjuvant CRT [39]. This includes the following tumor stages: (i) cT3a/b very low rectum with no involvement of the levator ani or mesorectal fascia (MRF negative), (ii) cT3a/b in mid‐ or high rectum, cN1‐2, no extramural vascular invasion (EMVI negative) and (iii) ≥cT3b and EMVI positive. Furthermore, they must be suitable to withstand the course of standard neoadjuvant CRT. Major exclusion criteria are metastatic disease, which is considered incurable by local therapies, as well as any medical contraindication for a standard neoadjuvant CRT or any other contraindication according to the IPI/NIVO SmPC.

### 4.3 Treatments (Neoadjuvant CRT, IPI, NIVO, Surgery)

External beam radiotherapy (EBRT) was delivered by volumetric‐modulated arc therapy (VMAT) and image guidance was done with cone beam computed tomography (CBCT). A total of 50 gray (Gy) was applied in 2 Gy fractions (over 25 working days) concomitant with capecitabine 1650 mg per m^2^ body surface area (BSA). A single dose of IPI (1 mg/kg) was administered intravenously (IV) over 30 min on Day 7, following three doses of NIVO (3 mg/kg) IV over 30 min every two weeks, starting on Day 14. Patients are planned to undergo laparoscopic, robotic, or open total mesorectal excision (TME) with a protective loop ileostomy within 10–12 weeks post‐CRT.

### 4.4 Myotoxicity Biomarkers

Skeletal muscle biomarkers (creatine kinase, CK; myoglobin, MB) and cardiac muscle biomarkers (cardiac troponin T, cTnT, Elecsys Troponin T‐high sensitive, Roche Diagnostics; creatine kinase‐muscle brain, CK‐MB; aspartate transaminase, AST; lactate dehydrogenase, LDH; N‐terminal prohormone brain natriuretic peptide, NT‐proBNP) biomarkers were measured at baseline, following weekly assessments until Week 6 and then continued in a 3‐week interval until surgery, as well as at the end of study visit (EOSV). Additional time points (outside the study protocol) were included when clinically needed or indicated. Biomarkers were routinely assessed by the Department of Laboratory Medicine of the Medical University of Vienna. High levels were defined as those higher than the upper limit normal (ULN) of the specific lab value. Cardiac troponin I (cTnI, ARCHITECT STAT High Sensitive Troponin‐I, Abbott) was analyzed from our prospectively biobanked patient plasma samples at the Department of Laboratory Medicine of the Medical University of Vienna.

### 4.5 Prospective Biobanking

Biomaterial (plasma) was prospectively processed and stored according to standard operating procedures of the Biobank of the Medical University of Vienna (ISO 9001:2015 certified) [40]. Biobanking timepoints included: baseline, Week 1, 2, 4, 6, the day before surgery, and the EOSV.

### 4.6 Myositis Antibodies

At the time of clinically suspected ICI‐induced myositis (CK and cTnT values >ULN) patients underwent assessment for myositis autoantibodies (Jo‐1, PM/Scl‐100, PL‐7, PL‐12, Mi‐2, Ku (p70/80), SRP) using line immunoassay (Department of Laboratory Medicine, Medical University of Vienna). In addition, patient samples were tested for myositis‐specific antibodies (MSA) and myositis‐associated antibodies (MAA) using the EUROLINE Autoimmune Inflammatory Myopathies 16 Ag (IgG) test kit (Division of Neuropathology and Neurochemistry, Department of Neurology, Medical University of Vienna). This assay encompassed the study following antigens: Mi‐2α, Mi‐2β, TIF1γ, MDA5, NXP2, SAE1, Ku, PM‐Scl100, PM‐Scl75, Jo‐1, SRP, PL‐7, PL‐12, EJ, OJ and Ro‐52.

### 4.7 Muscle Biopsies and Histopathological Work‐Up

Patients suspected of ICI‐induced myositis (CK and cTnT values >ULN with or without overt clinical symptoms) underwent muscle biopsy of their vastus lateralis muscle under local anesthesia. Freshly isolated muscle biopsies were transversally oriented, placed on a cork disk frozen in isopentane, and stored at −196°C in liquid nitrogen (Division of Neuropathology and Neurochemistry, Department of Neurology, Medical University of Vienna). About 8 µm thick frozen sections were obtained and were processed for histology and histochemistry, including the following stainings/reactions: hematoxylin‐eosin (HE), periodic acid‐Schiff (PAS) for glycogen, Oil Red O for lipids, Goemoeri trichrome and nicotinamide adenine dinucleotide (NADH) stain for mitochondria and sarcoplasmic reticulum, cytochrome C oxidase‐succinate dehydrogenase (COX‐SDH) stain for mitochondrial enzymes, myosin ATPase staining (at pH 4.3, 4.6 and/or 9.4) and antibodies directed against fast and slow myosin fibers and utrophin. Phenotypic characterization of muscle immune infiltrate was done by routine IHC using standardized protocols on a Dako Autostainer Link 48 (Division of Neuropathology and Neurochemistry, Department of Neurology, Medical University of Vienna). This included the following antigens: CD45, CD3, CD4, CD8, CD20, CD68, CD79a, HLA‐DR, C5b9, CD34, and p62 (Table S1). For electron microscopy (EM) samples were separately fixed in glutaraldehyde and embedded in Epon Resin. Semithin sections (1–2 µm) were stained with Toluidine blue (TBO) and thin sections (50–60 nm) were contrasted with uranyl acetate and lead citrate for further ultrastructural analysis.

### 4.8 Histopathological Assessment of Muscle Biopsies

We evaluated the presence, distribution, and severity of the following morphological features: Variations of muscle fiber size and shape with special attention on perifascicular distribution, fiber necrosis and their stages, myophagia, regenerating fibers, muscle fiber splitting, presence of ragged‐red fibers, vacuolar change, alterations of fat and/or glycogen content, density of capillaries and thickness of vascular wall, endomysial, perivascular, perimysial or epimysial cellular inflammatory infiltrates, and edema of connective tissue. We further characterized the muscle immune cell infiltrate (T and B cells) and the presence of membrane attack complex (MAC) in or around muscle fibers and capillaries. Following current concepts of idiopathic inflammatory myopathies (IIM) we categorized the myopathology as polymyositis (PM)‐like, dermatomyositis (DM)‐like, inclusion body myositis (IBM)‐like, immune‐mediated necrotizing myopathy (IMNM), and anti‐synthetase syndrome (AS)‐like [41, 42]. Semiquantitative evaluation was conducted using a multiheaded microscope and independently assessed by two expert neuropathologists (EG and SH), who were blinded to the clinical course, treatment and outcomes: muscle fiber necrosis (absent = 0, mild = 1, 1–2 necrotic fibers; moderate = 2, 3–10 necrotic fibers; severe = 3, >10 necrotic fibers), perifascicular atrophy or necrosis (absent = 0, present = 1), connective tissue edema (absent = 0, present = 1), presence of endomysial/perimysial infiltrates (absent = 0, mild = 1, moderate = 2, severe = 3). COX‐negative fibers (absent = 0, mild = 1, 1–2 negative fibers, moderate = 2, 3–10 negative fibers, severe = 3, >10 negative fibers), presence of rimmed vacuoles, as assessed by HE, Goemoeri and p62 IHC (absent = 0, present = 1), T cells (CD8), B cells (CD20), upregulation of HLA‐class I antigen and deposits of C5b9 (0 = absent, 1 = necrosis, 2 = capillaries, 3 = sarcolemmal (at least 2/3 of the sarcolemmal circumference granular staining), 4 = necrosis+sarcolemmal, 5 = necrosis+sarcolemmal+capillaries).

### 4.9 Whole‐Slide Tissue Cell Quantification of Muscle Biopsies

IHC muscle biopsy staining (CD4, CD8, CD20, CD45, and CD68) was absolutely quantified using a whole‐slide imaging approach. Briefly, microscopic whole‐slide images were acquired by the Vectra Polaris Automated Quantitative Pathology Imaging System (PerkinElmer, Hopkinton, MA, USA). Absolut positive cell count quantification was conducted with Multiplex IHC (v3.1.4) and a customized Nuclei Segmenter (HALO AI) module from the HALO Image Analysis Platform (Indica Labs, Albuquerque, NM, USA).

### 4.10 Analysis of Mismatch Repair (MMR) Status

MMR status was routinely assessed by IHC by the Department of Pathology, Medical University of Vienna. Additional details are available in the Supporting Information.

### 4.11 Tumor Mutation Analysis

Next‐generation sequencing (NGS) was carried out by the Department of Pathology, Medical University of Vienna. Additional details are available in the Supporting Information.

### 4.12 Statistical Analysis

Quantitative data were summarized using descriptive statistics (i.e. median, range, etc.) where applicable. No inferential statistical tests were performed, as the study was not designed for comparative or hypothesis‐driven analysis. All analyses were conducted with the primary aim of characterizing the observed patterns rather than testing predefined hypotheses.

## Author Contributions

J.L. and M.B. conceived and designed the CHINOREC study. R.Z., S.H., J.B.K., I.K., A.K., D.A., J.K., A.S., R.S., L.M., C.B., F.L., K.M., S.B., M.B., and J.L. acquired data for the work. R.Z., S.H., L.M., and J.L. performed analyses. R.Z., J.B.K., K.M., S.B., M.B., and J.L. interpreted the data. R.Z. and J.L. wrote the paper. R.Z., S.H., J.B.K., I.K., A.K., D.A., J.K., A.S., R.S., L.M., C.B., F.L., K.M., S.B., M.B., and J.L. have read and approved the final manuscript. Furthermore, R.Z., S.H., J.B.K., I.K., A.K., D.A., J.K., A.S., R.S., L.M., C.B., F.L., K.M., S.B., M.B., and J.L. authors advocate that any part of the present work was appropriately investigated and resolved. J.L. and M.B. were the guarantors. All authors have read and approved the final manuscript.

## Ethics Statement

The CHINOREC study protocol received ethical approval from the lead ethical committee (EC) of the Medical University of Vienna (No. 2040/2019) and was further endorsed by all local institutional review boards (IRB) of the participating centers. The study was carried out in consensus with the latest International Council for Harmonization of Technical Requirements for Pharmaceuticals for Human Use (IHC) Good Clinical Practice (GCP), the Declaration of Helsinki, as well as in alignment with the applicable local health authorities (HA). All active participants gave written informed consent before randomization.

## Conflicts of Interest

The investigator sponsored research (ISR) from Bristol‐Myers Squibb (BMS) was granted to J.L. and M.B. All other authors declare no competing interest regarding the present manuscript.

## Supporting information




**Supporting File 1**: Supplementary data final final.docx

## Data Availability

The data generated in this study are available within the article and its Supplementary Information. Data is available on reasonable request to the corresponding author with an appropriate IRB verification and a CDA. However, protected health information cannot be disclosed.
