# Supplementary material for: Incidence and Immunopathology of Myositis in Rectal Cancer Patients Treated With Neoadjuvant Immune Checkpoint Inhibitors and Chemoradiotherapy: Findings From the CHINOREC Trial
Source: MedComm (2020). 2025 Jul 7;6(7):e70275. doi: 10.1002/mco2.70275 (PMC12231051; doi:10.1002/mco2.70275)
Supplement: Supplementary file 1 — Supporting File 1: Supplementary data final final.docx [file MCO2-6-e70275-s001.docx]

**Incidence and immunopathology of myositis in rectal cancer patients treated with neoadjuvant immune checkpoint inhibitors and chemoradiotherapy: Findings from the CHINOREC trial**

Rebecca Zirnbauer^1^, Simon Hametner^2^, Jutta Bergler-Klein^3^, Irene Kuehrer^1^, Askin Kulu^1^, Daphni Ammon^1^, Julijan Kabiljo^1^, Anton Stift^1^, Rainer Schmid^4^, Leonhard Müllauer^5^, Clemens Bittermann^6^, Friedrich Laengle^6^, Klaus Machold^7^, Stephan Blüml^7^, Michael Bergmann^1,*^, Johannes Laengle^1^

^1^Division of Visceral Surgery, Department of General Surgery, Comprehensive Cancer Center Vienna, Medical University of Vienna, Waehringer Guertel 18-20, A-1090 Vienna, Austria

^2^Division of Neuropathology and Neurochemistry, Department of Neurology, Medical University of Vienna, Waehringer Guertel 18-20, A-1090 Vienna, Austria

^3^Division of Cardiology, Department of Medicine II, Medical University of Vienna, Waehringer Guertel 18-20, A-1090 Vienna, Austria

^4^Department of Radiation Oncology, Comprehensive Cancer Center Vienna, Medical University of Vienna, Waehringer Guertel 18-20, A-1090 Vienna, Austria

^5^Department of Pathology, Comprehensive Cancer Center Vienna, Medical University of Vienna, Waehringer Guertel 18-20, A-1090 Vienna, Austria

^6^Department of Surgery, State Hospital Wiener Neustadt, Corvinusring 3-5, A-2700 Wiener Neustadt, Austria

^7^Division of Rheumatology, Department of Medicine III, Medical University of Vienna, Waehringer Guertel 18-20, A-1090 Vienna, Austria

***Corresponding author**

Michael Bergmann, MD

Division of Visceral Surgery

Department of General Surgery

Comprehensive Cancer Center Vienna

Medical University of Vienna

Waehringer Guertel 18-20, 1090 Vienna, Austria

[michael.bergmann@meduniwien.ac.at](mailto:michael.bergmann@meduniwien.ac.at)

**Supplementary Figures**

**Figure S1: Study design of the CHINOREC trial.**

Overview of the CHINOREC trial, a prospective, randomized, open-label, multicenter, phase II investigator-initiated trial (IIT).

**Figure S2: Consort flow diagram.**

Diagram depicting patient allocation. CRT, chemoradiotherapy; IPI, ipilimumab; NIVO, nivolumab

**Supplementary Tables**

**Table S1. List of antibodies used for IHC**

| **Target** | **Host** | **Clone** | **Dilution** | **Company** |
| --- | --- | --- | --- | --- |
| CD3 | rabbit | A0452 | 1:100 | Dako |
| CD4 | mouse | 4B12 | 1:100 | Dako |
| CD8 | mouse | C8/114B | 1:400 | Dako |
| CD34 | mouse | NCL-L-END | 1:100 | Novocastra |
| CD20 | mouse | L26 | 1:2,000 | Dako |
| CD45 | mouse | 2B11+PD7/26 | 1:2,000 | Dako |
| CD68 | mouse | KP1 | 1:10,000 | Dako |
| CD79a | mouse | JCB117 | 1:200 | Dako |
| HLA-DR | mouse | CR3/43 | 1:400 | Dako |
| C5b9 | mouse | aE11 | 1:400 | Dako |
| p62 | mouse | 3/P62 lck ligand | 1:1000 | BD Bioscience |
| MLH1 | mouse | M1 | 1:100 | Ventana |
| MSH2 | mouse | G219-1129 | 1:100 | Cell Marque |
| MSH6 | mouse | 44 | 1:100 | Cell Marque |
| PMS2 | mouse | EPR3947 | 1:100 | Cell Marque |

**Supplemental Materials and Methods**

**Patients**

Detailed clinical case descriptions of ICI-induced myositis cases. Patient 1, has morbid obesity (BMI >40 kg/m^2^) and hypertension. The patient’s concomitant medications consisted of a statin (atorvastatin), an anti-platelet agent (acetylsalicylic acid), a beta blocker (bisoprolol), an angiotensin-converting-enzyme (ACE) inhibitor (enalapril) and a diuretic (hydrochlorothiazide). Shortly after the second dose of NIVO (22 days after first ICI dose) the patient developed a striking increase in myotoxicity biomarkers. At that time the patient however was asymptomatic and the ECG was normal. A coronary computed tomography (CT) angiography (CTA) determined an Agatston score of 1576 Hounsfield units (HU) in the right coronary artery (RCA) and left anterior descending (LAD) artery. Due to suspected myotoxicity, glucocorticoids (GC) were promptly initiated (methylprednisolone 2 mg/kg IV). The next day a transthoracic echocardiogram (TTE) was performed, which revealed a normal left ventricular ejection fraction (LVEF) without regional wall motion abnormalities. Due to continuously increasing myotoxicity biomarkers a coronary angiography was conducted, presenting only a non-significant stenosis (<30%) of the RCA. All other coronary arteries (left main, LAD and left circumflex) appeared to be normal without any signs of stenosis. Cardiac magnetic resonance (CMR) demonstrated no signs of myocarditis (LVEF 72%). Later that day the patient developed a mild right-sided ptosis and ipsilateral facial hypoesthesia. Consequently, a brain MRI was conducted, which displayed only a discreet and unspecific sulcal and leptomeningeal enhancement (LME). Cerebrospinal fluid (CSF) from a lumbar puncture demonstrated no specific pathological features. Except for anti-acetylcholine receptor (AChR) antibodies, all autoantibodies including the myositis panel were negative. The electromyography (EMG) was normal. An MRI of the pelvis and upper legs demonstrated a moderate signal alteration and edematous soaking of the adductor muscles, especially of the vastus lateralis muscle, congenial to an incipient myositis. Myotoxicity biomarkers declined under GC monotherapy. Apart from the modest right-sided ptosis, the patient remained asymptomatic and was discharged after approximately 2 weeks of diagnostic work up. Two weeks later the patient developed progressive muscle weakness and dyspnea requiring hospitalization. Intravenous immunoglobulin (IVIG) 2 g/kg was initiated. Repeated TTE was unchanged with no signs of LVEF dysfunction and the chest CT of the lungs was also normal. The patient’s vigilance progressively declined. The patient developed a CO_2_ narcosis requiring intubation and transfer to an intensive care unit (ICU). A muscle biopsy was taken from the vastus lateralis. Immediately, plasma exchange (PLEX) was initiated for 2 cycles, followed by infliximab (INFLIXI) 5 mg/kg IV. Two weeks later INFLIXI (5 mg/kg IV) was administered again, followed by 5 cycles of PLEX. After a challenging recovery phase and prolonged stay at the ICU the patient was eventually transferred to an intermediate care (IMC) and subsequently to the general ward. As a maintenance therapy he continued to receive IVIG (2 g/kg) every 4 weeks, while GC were slowly tapered over a 6-week period. After some weeks of prehabilitation the patient underwent delayed (24 weeks post CRT) but successful radical tumor surgery (R0) without any major surgical complications and could be discharged to proceed with further rehabilitation. He continued receiving IVIG monotherapy every 4 weeks as a maintenance therapy, which was later extended to an 8-week interval. By the time of the EOSV, AChR antibodies were no longer detectable. Currently, after 18 months of follow-up, the patient shows no signs of tumor recurrence and the age-appropriate instrumental and self-care activities of daily living (ADL) are unimpaired.

Patient 2 has no comorbidities or cardiovascular history and no concomitant medications, similarly, exhibited an increase in myotoxicity biomarkers shortly after the second dose of NIVO (23 days after first ICI dose). At that time the patient generalized weakness and the ECG normal. CMR and muscle MRI were discontinued by the patient due to anxiety attacks. A TTE revealed a normal LVEF without signs of wall motion abnormalities. All autoantibodies tested negative, including those in the myositis panel, except for anti-gastric parietal cell (GPC) antibodies. However, the patient showed no clinical signs of autoimmune gastritis and pernicious anemia. Simultaneously, he developed an ICI-induced thyroiditis with positive anti-thyroid peroxidase (TPO) and anti-thyroglobulin (Tg) antibodies. A muscle biopsy was taken from the vastus lateralis. Consequently, GC (prednisolone 1 mg/kg PO) treatment was initiated, followed by 3 cycles of PLEX and IVIG (2 g/kg). Remaining rather oligosymptomatic without limiting the instrumental ADL, the patient could be discharged from the hospital after myotoxicity biomarkers declined. IVIG was repeated once after 4 weeks, while GC was slowly tapered over 6 weeks. The patient underwent successful R0 tumor resection without surgical delay or complications.

Patient 3 has hypertension and coronary heart disease (CHD). The patient’s concomitant medications consisted of an ACE inhibitor (lisinopril) and a diuretic (hydrochlorothiazide). Soon after the second dose of NIVO (36 days after first ICI dose) the myotoxicity biomarkers increased. At that time the patient had generalized weakness, and the ECG was normal. CMR demonstrated no signs of myocarditis (LVEF 61%). Promptly, a muscle biopsy was taken from the vastus lateralis. All autoantibodies, including the myositis panel were negative. GC (prednisolone 1 mg/kg PO) treatment was initiated simultaneously to IVIG (2 g/kg). GC was tapered over 6 weeks and the patient received the scheduled oncological tumor resection (R0) without surgical delay or major complications.

Patient 4 has no comorbidities or concomitant medications. After the second dose of NIVO (28 days after first ICI dose) the patient presented increased myotoxicity biomarkers. Initially the patient had generalized weakness relieved by rest. ECG, TTE and CMR (LVEF 66%) were inconspicuous for signs of cardiac involvement. GC (prednisolone 1 mg/kg PO) and IVIG (2 g/kg) treatment was promptly initiated. A muscle biopsy from the vastus lateralis was performed. All autoantibodies were negative. The weakness and muscle pain subsided with the decline of myotoxicity biomarkers. The GC was tapered over 4 weeks, and IVIG (2 g/kg) was administered once more 4 weeks after the initial dose. Unfortunately, the patient began exhibiting symptoms again and the levels of myotoxicity biomarkers started to rise once more. INFLIXI (5 mg/kg IV) was initiated and repeated after 2 weeks. IVIG (2 g/kg) was also repeated 4 weeks after the last application. Finally, GC could be tapered and the patient underwent successful tumor resection (R0) without any delay or any surgical complications.

Patient 5 has hypertension and is taking an angiotensin II receptor blocker (candesartan). The first dose of NIVO was postponed for 2 weeks, due to COVID-19 (asymptomatic, only PCR positive). Soon after the second dose of NIVO (56 days after first ICI dose) the patient presented with increased myotoxicity biomarkers. At this time, the patient had generalized weakness relieved by rest. ECG, CMR (LVEF 62.3%), TTE and coronary CTA (CAD-RADS 1) were all normal. GC (prednisolone 1 mg/kg PO) and IVIG (2 g/kg) treatment was promptly initiated. Muscle biopsy of the vastus lateralis was done. All autoantibodies were negative. Remaining rather oligosymptomatic GC was tapered. However, due to ICI-induced hepatitis (92 days after first ICI dose) GC dose needed to be increased again (prednisolone 60 mg PO). Repeated measuring of autoantibodies was still negative. The re-staging (DRE, endoscopy, biopsy, CEA, PET MRI) demonstrated a clinical complete response (cCR). The patient chose a watch-and-wait strategy (outside of the study protocol). In the 2 months that followed, the patient progressively developed a peripheral sensory neuropathy of the lower and upper limbs, Bell's palsy (left side) and the generalized weakness progressed in terms of limiting the instrumental and self-care ADL. GC (prednisolone 1 mg/kg PO) were again initiated, which remitted the Bell's palsy but not the generalized weakness. The first follow-up at 2 months however showed stable cCR. Due to progressive symptoms, the patient was hospitalized for further diagnostic work up. AChR antibodies, EMG and repeated TTE were all normal. Nerve conduction velocity (NCV) suggested an axonal sensorimotor polyneuropathy of the lower extremities. Repetitive stimulation was conspicuous, albeit inconclusive (pathological decrement before volitional activation, which was no longer reproducible after volitional activation). MRI of the cranium and cervical spine was normal (besides the suspicion of a slight neuritis her right facial nerve). IVIG (2 g/kg) was repeatedly applied. The patient’s symptoms improved slightly so he was discharged from hospital. GC were further denied by the patient and an attempt with pyridostigmine was agreed on. However, this did not really improve much of the patient’s symptoms and GC (prednisolone 1 mg/kg PO) was reintroduced and tapered over 8 weeks. The patient symptoms improved steadily over time and the age-appropriate instrumental and self-care ADL are almost unimpaired. The second follow-up at 5 months was suspicious for a local re-growth. Due to the patient wish, a sphincter-sparing surgery in terms of a transanal excision (TAE) was successfully performed (R0) without any surgical complications.

Patient 6 has no comorbidities or concomitant medications. After the second dose of NIVO (35 days after first ICI dose) the patient had increased myotoxicity biomarkers. The patient had generalized weakness, which was relieved by rest. ECG, CMR (LVEF 58.8%), TTE and coronary CTA (LAD and RCA with an Agatston score 93) were all normal. All autoantibodies were negative. GC (prednisolone 1 mg/kg PO) and IVIG (2 g/kg) treatment was promptly initiated. A muscle biopsy from the vastus lateralis was performed. Myotoxicity biomarkers declined and GC was tapered over 4 weeks. The generalized weakness however did not really improve and shortly before the scheduled tumor surgery the patient’s levels of myotoxicity biomarkers started to rise once more. GC (prednisolone 1 mg/kg PO) was reintroduced for 3 days. Myotoxicity biomarkers declined and the patient received an oncological tumor resection (R0), unfortunately with a major surgical complication (anastomotic leak) and a prolonged hospitalization with Endo-VAC. Finally, he could be discharged, without any recurrent symptoms form the myositis.

** Proposed screening and treatment algorithm for ICI-induced myotoxicities**

ECG, electrocardiogram; TTE, transthoracic echocardiogram; CMR, cardiac magnetic resonance; ICI, immune checkpoint inhibitors; coronary CTA, computed tomography angiography; CK, creatine kinase; cTnT, cardiac troponin T; cTnI, cardiac troponin I; GC, glucocorticoids; INFLIXI, Infliximab; PLEX, plasma exchange; IVIG, intravenous immunoglobulin; CRT, chemoradiotherapy

**Analysis of mismatch repair (MMR) status**

MMR status was routinely assessed by IHC (Department of Pathology, Medical University of Vienna). Briefly, formalin-fixed paraffin-embedded (FFPE) pre-treatment tumor biopsies and/or resected tumor specimen were IHC-stained for MLH1, MSH2, MSH6 and PMS2 using routine protocols on a VENTANA BenchMark ULTRA immunostainer (**Supplementary Table 1**). Loss of protein expression was classified as deficient MMR (dMMR). Matched normal tissue served as an internal positive control.

**Tumor mutation analysis**

Next generation sequencing (NGS) was carried out with an Ion GeneStudio™ S5 System (Thermo Fisher Scientific Inc., Waltham, MA, USA) according to manufactures protocol (Department of Pathology, Medical University of Vienna). Patient’s FFPE tumor tissue was used for amplicon sequencing. The Ion AmpliSeq™ Colon and Lung Cancer Research Panel v2 includes the following genes: *KRAS, EGFR, BRAF, PIK3CA, AKT1, ERBB2, PTEN, NRAS, STK11, MAP2K1, ALK, DDR2, CTNNB1, MET, TP53, SMAD4, FBX7, FGFR3, NOTCH1, ERBB4, FGFR1* and *FGFR2.* The sequences were analyzed with Ion Reporter™ Software (Thermo Fisher Scientific Inc.).
